# Tr. Ferri Mur.

**Published:** 1879-11

**Authors:** 


### TR. FERRI MUR.

DURING the administration of the tincture of the chloride of iron, functional derangements of the stomach and liver often arise, with furred tongue, impaired appetite, headache, etc. These symptoms rapidly disappear upon adding one-half grain of the chloride of ammonia to each minim of the tincture. This combination is useful in cases of heart disease, accompanied by anæmia and debility.—*Boston Medical and Surgical Journal.*

